# Integrating traditional and herbal medicine into heatwave resilience and care

**DOI:** 10.3389/fphar.2025.1712917

**Published:** 2026-01-09

**Authors:** M. L. Manoj, Kranthi Kiran Akula, Harsha Hegde, Subarna Roy

**Affiliations:** Indian Council of Medical Research‐National Institute of Traditional Medicine (ICMR-NITM), Belagavi, Karnataka, India

**Keywords:** climate change adaptation, heat action plan, heat resilience, heat stress, heat-related illnesses, traditional medicine

## 1 Introduction

Human-induced emissions are slowly pushing average global temperatures to new heights. Most outdoor workers, such as farmers, construction workers, delivery workers, and street vendors, cannot avoid heat exposure, especially during heat waves. Excessive exposure to heat renders people vulnerable to heat-related illnesses, worsens the underlying heart and kidney diseases, and strains health services (Haines et al., 2006). A study found more than a 30% increase in mortality on days with temperatures higher than 40 °C compared to days where the temperature is ≤ 38 °C in northern India. It is projected for India that a 2 °C increase in the average temperatures can substantially increase mortality among the elderly above 65 years by mid-21st century (Agarwal et al., 2018; Romanello et al., 2023).

There is growing evidence for the effectiveness of established traditional medicines (TM) and herbal medicines (HM), which have been in use in India for centuries to manage heat-related illnesses and better heat adaptation. We opine that integrating TMs and HMs alongside the standard guidance, such as shade, safe water/ORS, rest, rapid cooling, and early referral, can help against heat-related illnesses by reducing the effects of the heat stress and aiding in faster recovery to make people more resilient. The reason why this method is realistic is that a large number of communities in India already adhere to seasonal nutrition plans, herbal remedies and behavioral routines discussed in Ayurveda and other Indian traditional systems. At the same time, stringent clinical assessment and standardization of these practices is necessary as the number of heat events is on the rise and vulnerable populations, including outdoor workers, the ageing, and the urban poor, are subjected to increased risks (Haines et al., 2006; Romanello et al., 2023).

## 2 What TM/HM can offer-and what it cannot

### 2.1 Prevention and preparedness

In India, the NDMA (NDMA, 2019) guidelines (2019) and the Ministry of Health and Family Welfare’s National Action Plan for Heat-Related Illnesses (MoHFW, 2021) are key frameworks for seasonal heat emergency preparedness and treatment of heat-related health issues. Seasonal guidance with respect to lifestyle changes in TM systems, such as Ayurveda, is found to overlap with heat preparedness, which includes lighter meals, regular small sips of fluids, and familiar cooling foods/drinks. During the summer months, it is a common practice in India for people to take tender coconut water after exhaustion, or to take buttermilk, lemon–salt water, cucumber, *Hemidesmus indicus* (L.) R.Br. [Apocynaceae] (Indian sarsaparilla) root extract, vetiver root extract, etc., for rehydration; some of these also help with electrolyte replenishment. These are low-cost, locally available, and easy to adopt, and have greater compliance rates. Ayurveda recommends eating foods with “*sheeta veerya”* or “cool potency”, which are light, easy to digest, and cooling to better adapt to summer heat. In this framework, foods and drinks dominated by the water element (*jala*) and described as cooling - such as tender coconut water, diluted buttermilk, fresh seasonal fruits (for example, watermelon, muskmelon, grapes and pomegranate) and cucumber preparations - are traditionally used to counteract excess internal heat and dryness. These are typically mild in taste, non-greasy and easy to digest, and they align closely with modern advice to increase fluid intake and consume high-water content foods in summer (Saini, 2018; Nepal et al., 2023).

### 2.2 Early symptom relief and recovery support

There are established traditional practices for treating various heat-related illnesses. Traditional topical remedies like Aloe gel, sandalwood paste, and rose water are commonly used for prickly heat and irritated skin, giving relief and helping people tolerate hot and humid conditions better (Saini, 2018; Safia et al., 2019; Maan et al., 2018). Breathing practices such as ‘*Sitali’* and ‘*Sitkari’*, along with short yoga routines that can be nested under mind-body interventions, are known to cool down the body and improve comfort and sleep. These practices can act as adjuncts that make it easier to follow the basics - drinking fluids, resting, and staying cool. They can also support quicker recovery after being affected by heat-related illnesses (Yadav et al., 2019; Telles et al., 2020; Estevao, 2022). While helpful, they are not treatments for heat stroke but supportive measures that can add comfort and aid in recovery.

### 2.3 Possible pre-conditioning and stress modulation

Many medicinal plants used in TM systems are recommended to be used during the hot season to treat heat-related illnesses. *Pitta* is one of the three fundamental energies (*doshas*) in Ayurveda and is primarily associated with the fire element and with metabolic heat, digestion and transformation. Hot environments, intense physical exertion, irregular sleep and intake of alcohol and extremely spicy/sour foods are understood to aggravate *Pitta*, predisposing individuals to symptoms such as burning sensations, dizziness, irritability, headache and disturbed sleep during hot seasons. In this sense, classical summer regimens (*Greeshma ritu charya*) can be interpreted as a set of dietary and lifestyle measures that prevent *Pitta* aggravation and support hydration and cooling, complementing public health advice on shade, fluids and rest (Saini, 2018; Nepal et al., 2023). Adaptogenic plants that are described as stress-protective, such as *Withania somnifera* (L.) Dunal [Solanaceae] (ashwagandha), *Tinospora cordifolia* (Willd.) Miers [Menispermaceae] (guduchi), and *Asparagus racemosus* Willd. [Asparagaceae] (shatavari) help the body to resist stress and maintain a balance; recent research work points to mechanisms acting on the Hypothalamic-Pituitary-Adrenal (HPA) axis, potentially activating heat-shock responses (HSP70/HSP90), antioxidant and anti-inflammatory pathways (Panossian et al., 2021; Singh et al., 2024). These mechanisms are promising, but there is a need for pragmatic trials that show fewer heat-symptom days, fewer emergency visits, and less work loss before any policy changes are made.

### 2.4 Hydration and electrolytes

To address dehydration, which in turn leads to electrolyte loss from heat exposure, drinking electrolyte-rich supplements works well (Casa et al., 2000). Salted buttermilk and tender coconut water are common traditional choices during the summer months. When given as an adjunct during heat stress, tender coconut water rich in potassium and magnesium supports post-exertion recovery (Prades et al., 2012; O’Brien et al., 2023). Other traditional drinks rich in electrolytes include amla juice, raw mango drink and tamarind pulp drink. They are good rehydration and electrolyte replenishment options when prepared hygienically. But caution is needed regarding toxicity related to overconsumption because some of these drinks, such as coconut water or watermelon, are high in potassium. Community advice should include warnings for people with chronic kidney disease (CKD), heart failure, or those taking ACE inhibitors (Prabhu et al., 2024).

### 2.5 What traditional medicine (TM)/herbal medicine (HM) cannot do

TM/HM is not a substitute for prevention or clinical care. Severe heat exhaustion or any suspicion of heat stroke needs rapid cooling of the patient’s body and also requires urgent referral (Hifumi et al., 2018; Sorensen and Hess, 2022). Traditional options must never cause delay in escalation. The clear and consistent message is simple: they are adjuncts, not replacements. The scope of TMs/HMs lies in prevention, mild symptom relief, and recovery, but not in emergency care.

## 3 A practical, integrated approach that fits public-health systems

### 3.1 Trigger and timing

Most cities and many countries issue heat alerts using local thresholds. In India, the National Disaster Management Authority (NDMA) (NDMA, 2019) gives the guidelines and advisories for countering heat waves and defines the thresholds. Along with the preparatory guidelines for countering heat waves, a short, safe, and practical TM/HM adjunct toolkit can be built, which can help make the vulnerable people more resilient and can also support treatment and recovery from heat-related illnesses. But a time-bound adoption should be ensured from pre-alert until the end of the heat spell; this is pragmatic and also lowers the risk that people may not overuse or consume remedies without supervision.

### 3.2 An alert-ready adjunct toolkit for indian conditions

In addition to core measures such as access to safe drinking water, ORS, shaded or cooled spaces, and timely medical referral, simple culturally familiar adjuncts can make heat preparedness messages more acceptable and actionable in Indian settings. For this purpose, we propose an “alert-ready adjunct toolkit” that can be integrated into heat action plans (Table 1).

**TABLE 1:** An alert-ready adjunct toolkit for Indian conditions.

Intervention type	Intervention	Precautions	References
Hydration and electrolytes	• Safe drinking water and ORS as the base; in healthy individuals • Tender coconut water can be taken after exertion	Tender coconut water should be avoided or restricted in patients with chronic kidney disease (CKD) or hyperkalaemia, and this should be clearly stated in local guidelines	Singh et al. (2024), Prades et al. (2012)
Cooling topicals	• *Aloe vera* (L.) Burm.f. [Asphodelaceae] gel, rose-water rinses, and sandalwood paste can be used for local cooling and comfort after heat exposure	Do not apply on broken, blistered, or infected skin; in case of severe skin injury, immediate medical care should be sought. Stop use if any burning, rash, or other allergic reaction occurs	Saini, 2018; Safia et al. (2019), Maan et al. (2018)
Dietary support	• Amla and other antioxidant-rich seasonal fruits, light non-greasy meals, and vetiver root drinks as dietary support	Use fruits and sweetened drinks with caution in people with diabetes; ensure good-quality raw materials and hygienic preparation to avoid contamination or adulterated products	Saini (2018)
Recovery support	• 5–10 min of Sitali/Sitkari breathing exercises after heat exposure • Short, gentle evening yoga routine for sleep and stress regulation once the person is cooled and rehydrated	These practices are not a substitute for emergency treatment in moderate or severe heat illness. Avoid in people with acute respiratory distress, uncontrolled asthma, or when the person is confused or unstable	Yadav et al. (2019), Telles et al. (2020)
Emergency escalation	• If danger signs such as confusion, disorientation, unusual behaviour, fainting, seizures, inability to drink, persistent vomiting, extremely hot or dry skin, or very fast breathing/heart rate are present, treat as a medical emergency with rapid cooling and urgent referral	This is a mandatory action. Move the person to a cool place, start rapid cooling and arrange urgent transport to the nearest health facility	Hifumi et al. (2018), Sorensen and Hess (2022)

### 3.3 Delivery channels and messages

Use delivery channels that already reach high-risk groups. Primary-care staff, community health workers, self-help groups, worker associations and local governments and panchayats can deliver the required messages. Employer toolboxes in agriculture, construction, logistics, and factories can also include practical steps. Pair every “dos” list with a short “do not use” list and a red-flag graphic for escalation. Use regional languages and keep the visual infographics simple.

### 3.4 Why does the use of TMs/HMs improve equity?

For those most at risk and vulnerable, the challenge is not only a lack of knowledge but also the feasibility of adopting preventive measures. Often, outdoor workers have little flexibility in their working hours, and many households lack cooling technology. Using locally available foods and adopting familiar practices can increase compliance and adherence. Additionally, this approach will strengthen vulnerable populations against heat-related adverse events. It is to be noted that in an Indian setting, traditionally, the majority of men have outdoor occupations compared to women, making them vulnerable to heat-related illnesses.

### 3.5 Safety, quality, and regulation: non-negotiable guardrails of TM/HM

Public programs with TM/HM must follow a quality-before-quantity rule. Procurement of materials should only be done from GMP-compliant suppliers. Pharmacopeial identity tests and Certificates of Analysis for microbial load and heavy metals are also important. Labels must be clear, in local languages, with doses and cautions. Plain-language contraindication flags should be printed on every sheet: liquorice in hypertension, CKD, or heart failure; high-potassium drinks in CKD or with Renin-Angiotensin-Aldosterone System blockade medication, unsweetened options for diabetes. Also, it is essential to avoid plants listed in Schedule E(I) in the Drugs and Cosmetics Rules, 1945 (India), as they are potentially toxic.

### 3.6 Biological plausibility

TM may act on heat-related injuries by several mechanisms. To cope with heat exposure, the body produces sweat to cool down, leading to the loss of fluids/electrolytes, and if not replenished, will lead to cardiovascular and thermal strain (Casa et al., 2000). Including antioxidants and anti-inflammatory botanical drugs or supplements in the diet can help reduce oxidative stress and may lower the chance of heat-related injury. Plants with adaptogenic properties may modulate HPA-axis responses and also influence heat shock protein systems, which have a key role in adaptation to heat stress (Singh et al., 2024; Panossian and Wikman, 2010). However, the results from previous studies, while generally coherent, are largely derived from traditional/historical use along with evidence from preclinical models. However, there are relatively few well-designed clinical trials. It is therefore important to distinguish which interventions are supported mainly by traditional or preclinical evidence and which are backed by modern clinical data, in order to appropriately manage reader expectations.

### 3.7 What evidence do we further need for fast, pragmatic, and fair adoption?

Adopting established traditional practices requires filling key evidence gaps. Rapid, real-world data from simple field studies and implementation assessments can guide safe, practical, and equitable use.

1. Alert-aligned cluster trials (2–8 weeks): Randomize wards or worksites to “TM Toolkit + standard Heat-Health Action Plan (HHAP)” versus “standard HHAP” ensuring that all standard treatment protocols are strictly followed. Track all heat-symptom days per person, Emergency department visits, and missed work hours, along with all possible safety markers.
2. Adaptive trials for priority botanicals/formulations: They can be started with a standardized shortlist such as *Phyllanthus emblica L.* [Phyllanthaceae] (fruit in high vitamin C and polyphenol content, together with demonstrated antioxidant, anti-inflammatory, endothelial-protective properties and cardiometabolic benefits, it is a strong candidate for improving resilience and recovery in heat-exposed individuals) (Saini et al., 2022), *Chrysopogon zizanioides (L.) Roberty* [Poaceae] (root extracts have shown anti-inflammatory, analgesic, antioxidant and organ-protective activity, and their traditional use as cooling infusions during summer supports evaluation as symptom-relief and recovery adjuncts) (Gunasekar et al., 2025) and *Tinospora cordifolia* (It has immunomodulatory, hepatoprotective and antioxidant actions that may help mitigate systemic inflammation and organ stress during and after heat exposure, but its inclusion in public programmes must incorporate strict dose standardisation, exclusion of high-risk individuals and active monitoring for potential liver injury) (Chaudhary et al., 2024; Kulkarni et al., 2022). Endpoints can be core temperature recovery, sweat rate, and symptom burden, with multiple parameters that may suggest the underlying mechanisms. (Singh et al., 2024; Guo et al., 2021).
3. Implementation science: Compare community-led and employer-led delivery. Track uptake, adherence, acceptability, cost-effectiveness, and equity by gender and occupation.
4. Quality and pharmacovigilance systems. Set up sentinel clinics to capture adverse events and herb-drug interactions. Run batch audits for identity, contaminants, and stability for any publicly distributed product.
5. Community-based trials of heat-health education (with TM/HM adjuncts). Test a brief, culturally tailored education package. Include red-flag symptoms, when to escalate, safe fluids, simple TM/HM adjuncts, and a “do-not-use” list.

### 3.8 Limitations

There is not much heterogeneous evidence to support many plant-based options discussed. Preclinical doses and preparations differ greatly, and most of the evidence remains at the preclinical stages. Even in the limited cases of human studies, they are seldom aimed at the outcome of heat-specificity.

The availability and prices of TM/HM vary in different regions, and informal markets might provide uneven quality. This is why public programs should only procure vetted products and give clear guidance on what not to buy.

The knowledge of traditional medicine or local practices can also change with the region. Because of this, Toolkits may be made specific to regions (Hifumi et al., 2018; Sorensen and Hess, 2022).

## 4 Conclusion

The current trend in global emissions suggests heatwaves will intensify in the future. Combining traditional/herbal medicine with standard guidelines and using TMs/HMs as supplements in an integrative way can assist the population to prepare, minimize the effects of heat stress and recover quicker. The solution in our opinion is realistic: activate short, safe, culturally familiar toolkits when alerts are issued, which is effectively integrated in line with current guidelines and treatment protocols and is never recommended as a replacement to standard treatment. Generating evidence from pragmatic trials is essential in addressing the gaps.
